# Rescue and characterization of recombinant cedar virus, a non-pathogenic *Henipavirus* species

**DOI:** 10.1186/s12985-018-0964-0

**Published:** 2018-03-27

**Authors:** Eric D. Laing, Moushimi Amaya, Chanakha K. Navaratnarajah, Yan-Ru Feng, Roberto Cattaneo, Lin-Fa Wang, Christopher C. Broder

**Affiliations:** 1 Department of Microbiology and Immunology, Uniformed Services University, Bethesda, MD 20814 USA; 2 Department of Molecular Medicine, Mayo Clinic, Rochester, MN USA; 3 Programme in Emerging Infectious Diseases, Duke-NUS Medical School, Singapore, Singapore

**Keywords:** Cedar virus, Henipaviruses, *Paramyxoviridae*, Ephrin ligands, Reverse genetics, Recombinant virus, Receptor tropism

## Abstract

**Background:**

Hendra virus and Nipah virus are zoonotic viruses that have caused severe to fatal disease in livestock and human populations. The isolation of Cedar virus, a non-pathogenic virus species in the genus *Henipavirus*, closely-related to the highly pathogenic Hendra virus and Nipah virus offers an opportunity to investigate differences in pathogenesis and receptor tropism among these viruses.

**Methods:**

We constructed full-length cDNA clones of Cedar virus from synthetic oligonucleotides and rescued two replication-competent, recombinant Cedar virus variants: a recombinant wild-type Cedar virus and a recombinant Cedar virus that expresses a green fluorescent protein from an open reading frame inserted between the phosphoprotein and matrix genes. Replication kinetics of both viruses and stimulation of the interferon pathway were characterized in vitro. Cellular tropism for ephrin-B type ligands was qualitatively investigated by microscopy and quantitatively by a split-luciferase fusion assay.

**Results:**

Successful rescue of recombinant Cedar virus expressing a green fluorescent protein did not significantly affect virus replication compared to the recombinant wild-type Cedar virus. We demonstrated that recombinant Cedar virus stimulated the interferon pathway and utilized the established Hendra virus and Nipah virus receptor, ephrin-B2, but not ephrin-B3 to mediate virus entry. We further characterized virus-mediated membrane fusion kinetics of Cedar virus with the known henipavirus receptors ephrin-B2 and ephrin-B3.

**Conclusions:**

The recombinant Cedar virus platform may be utilized to characterize the determinants of pathogenesis across the henipaviruses, investigate their receptor tropisms, and identify novel pan-henipavirus antivirals. Moreover, these experiments can be conducted safely under BSL-2 conditions.

## Background

Hendra virus (HeV) and Nipah virus (NiV) are the prototypical viruses of the genus *Henipavirus* [1], and are notable highly pathogenic zoonotic paramyxoviruses that have caused numerous severe and often fatal acute respiratory and/or neurologic disease in humans and livestock since their initial recognition in Australia (HeV) and Malaysia, Bangladesh, India and the Philippines (NiV) (reviewed in: [2–4]). The identification of HeV following fatal respiratory illness in 17 horses and one human in 1994 [5], was followed by the first recorded spillovers of NiV between 1998 and 1999 in Malaysia, and subsequently Singapore, which caused cases of severe illness in pigs that was also transmitted to farmers and abattoir workers [6–11]. A genetically distinct but closely related strain of NiV has been responsible for febrile illness in India and annual outbreaks of acute encephalitis in Bangladesh with notable increased pathogenicity compared to the NiV-Malaysia strain: fatality rates 70–100% [12–14]. High pathogenicity and mortality rates associated with HeV and NiV infection have resulted in the classification of both viruses as select agents, and represent the only paramyxoviruses that require biological safety level 4 (BSL-4) containment [15].

The henipaviruses encode two envelope glycoproteins, attachment (G) and fusion (F) glycoproteins, which mediate pH-independent cellular attachment, fusion and virus entry. The functional receptors for HeV and NiV are the highly conserved receptor tyrosine kinase ephrin-B class ligands, ephrin-B2 (EFNB2) and ephrin-B3 (EFNB3) [16–20]. EFNB2 is expressed on vascular endothelial cells and in the brain [21, 22] and both HeV and NiV display a tropism for endothelial and neuronal tissue [23–25], while EFNB3 is more prominent in the brain and brainstem [22, 25, 26]. The conserved homology of EFN ligands is thought to facilitate the broad natural and experimental species tropism [10, 27–31], and the physiological distribution of EFNB2 and EFNB3 correlates with pathological outcomes of HeV and NiV infection such as vasculitis, central nervous system involvement and systemic dissemination [23–25]. In addition, a more efficient use of EFNB3 by NiV compared to HeV as an alternative receptor has been suggested to contribute to the observed increased likelihood of meningitis and encephalitis with NiV infection [32].

Bats in the genus *Pteropus* were identified as the natural reservoirs of HeV and NiV [33–36] and HeV and NiV transmission and spillovers correlated with *Pteropus* geographical distributions [2, 37, 38]. Evidence of henipaviruses has been detected in *Pteropus lylei* populations in Southeast Asia [39, 40], *Pteropus vampyrus* in Indonesia [41] and bat populations endemic to the African continent [42–45] indicative of a global distribution of henipaviruses. Genetic sequences corresponding to new *Henipavirus* species have been detected in bats endemic in both Africa and Central America [46, 47] and the nearly complete genome of one African henipavirus, *Ghanaian bat henipavirus* Kumasi Virus (KumPV) has been sequenced [47]. One exception to the preponderance of evidence that *Pteropid* bats are the natural hosts of henipaviruses, was the detection of *Mojiang henipavirus* (MojPV) sequences from a rodent host in China [48].

In 2012, Cedar virus (CedPV), a non-pathogenic *Henipavirus* species was isolated from urine collected under a roost of *P. alecto* and *P. poliocephalus* in Australia [49]. Genomic analysis revealed that CedPV was closely related to HeV and NiV, but was distinct in its use of EFNB2, but not EFNB3 for cellular entry, and lack of pathogenicity in animal models of infection [49]. In contrast to HeV and NiV, and nearly all other paramyxoviruses, the CedPV phosphoprotein (P) gene does not undergo RNA editing and does not produce the V or W proteins [49, 50]. Both HeV and NiV V and W proteins are potent antagonists of the toll-like receptor signaling and interferon (IFN) pathways [51–56], whereas the IFN response was not antagonized by CedPV infection [49, 50]. The key role of V protein in pathogenicity was demonstrated by a recombinant NiV, which resulted in a non-lethal, replication competent infection when the V protein was removed [57]. Taken together, the lack of V and W protein expression and inability to utilize EFNB3 could be suggestive of the failure of CedPV to cause clinical disease in animal infection models [49].

Without functional studies the pathogenic potential of novel and related henipaviruses remains obscure, and outside of HeV and NiV, CedPV remains the only documented *Henipavirus* species isolated. Because CedPV was isolated in a BSL-4 facility it cannot be removed and transferred to a lower containment laboratory. To develop a platform to understand pathogenesis of henipaviruses, we used a reverse genetics approach to rescue replication-competent, recombinant CedPV (rCedPV). Reverse genetic systems have been utilized for the generation of recombinant infectious and replication-competent negative sense RNA viruses with specific mutations and insertions [58, 59], particularly NiV and HeV [60–64]. Introduction of reporter genes, such as green fluorescent protein (GFP) or luciferase, provides for an ability to monitor virus replication and spread in real time and/or to perform high-throughput screening [63]. In this study, we describe the rescue of two rCedPV variants, one recombinant wild-type CedPV (rCedPV-wt) and one of which expresses GFP from an additional open reading frame (rCedPV-GFP). We compared the replication kinetics of both rCedPV variants and observed no significant differences between rCedPV-wt and rCedPV-GFP and characterized the ability of rCedPV to activate the IFN pathway. We confirmed that both rCedPV-wt and rCedPV-GFP utilized EFNB2 but not EFNB3 for cellular entry, but also observed rCedPV-GFP replication in cells previously characterized as EFNB2 negative.

## Methods

### Cells

BSR-T7/5, Vero E6, HeLa-USU, and HeLa-CCL2 (ATCC) cell lines were maintained in Dulbecco’s modified eagle media (DMEM) (Quality Biological; Gaithersburg, MD) supplemented with 10% cosmic calf serum (CCS) and 1% L-glutamine (Quality Biological; Gaithersburg, MD). HeLa-USU-ephrinB2 (EFNB2) and HeLa-USU-ephrinB3 (EFNB3) stable cell lines were maintained in DMEM 10% CCS, 1% L-glutamine supplemented with 0.4 mg/mL Hygromycin B (Invitrogen; Carlsbad, CA).

### Generation of plasmids and rescue of recombinant CedPV

Recombinant CedPV laboratory manipulation guidelines and standard operating procedures under BSL-2 conditions were developed and this work was reviewed and approved by the Uniformed Services University, Institutional Biosafety Committee in accordance with NIH guidelines. To construct the rCedPV antigenome clone, large DNA fragments of CedPV based on the Cedar virus isolate CG1a sequence, NCBI Accession number NC_025351.1 were synthesized (GenScript; NJ, USA). These DNA fragments corresponded to CedPV nucleotide bases: 1–4530, 4531–10,517, and 10,518–18,162. These fragments were sequentially cloned into an expression plasmid, pOLTV5 [65], between the T7 RNA promoter and hepatitis delta virus (HDV) ribozyme. The pOLTV5 vector was similarly used for cloning and expression of recombinant HeV [63]. At the time of the rCedPV cDNA clone design, the nucleotide at position 7 in the CedPV reference genome was a cytosine, which was later revised to an adenine. Our rCedPV cDNA clones have the cytosine at position 7. Furthermore, internal SmaI restriction sites in the CedPV reference genome were removed (C395A and C4816A) in the rCedPV cDNA clone to preserve the cloning strategy. A MluI restriction site was created between the P and M genes at nucleotide position 4531, after the M transcriptional start sequence, to facilitate insertion of a modified turbo Green Fluorescent Protein (GFP) gene (Lonza Inc., Allendale, NJ) [66]. To insert the GFP gene into the rCedPV antigenome plasmid, CedPV untranslated regions: transcriptional P stop, intergenic region, and transcriptional M start sequences (TAAGAAAAAACTTAGGATCCCAG) were amplified by polymerase chain reaction and cloned into the 3′ terminus of the GFP gene with an additional non-coding 3′ thymine nucleotide to maintain the “rule of six”. As noted in the rescue of recombinant HeV [63] and in contrast to the rescue of a recombinant NiV-GFP [60], GFP was inserted between the P and M genes to maintain the level of N and P necessary for proper virus replication. To generate replication helper plasmids, polymerase chain reaction was used to amplify the open reading frames of N, P, and L genes from rCedPV DNA plasmids, which were subsequently cloned into expression vectors that contain a cytomegalovirus promoter (pCMV) [67]. All plasmids were sequenced to obtain at least 2-fold sequence coverage. We adopted reverse genetic methods previously used to rescue recombinant HeV and NiV reporter viruses to generate rCedPV [60, 63].

To generate rCedPV-GFP and rCedPV-wt, pCMV-CedPV-N (1.25 μg), pCMV-CedPV-P (0.8 μg), pCMV-CedPV-L (0.4 μg), and pOLTV5 full-length CedPV antigenome plasmid (3.5 μg) were mixed with 12 μL of Lipofectamine LTX (Invitrogen; Carlsbad, CA) in 500 μL OptiMEM (GIBCO; Gaithersburg, MD) and incubated for 30 min at room temperature. This mixture of plasmid DNA and Lipofectamine LTX was used to transfect 5 × 10^5^ BSR-T7/5 cells. Four days post-transfection, GFP was observed in BSR-T7/5 cells transfected with the rCedPV-GFP antigenome and N, P, and L helper plasmids. Adherent BSR-T7/5 cells were collected along with culture supernatant and subjected to three rounds of freeze-thaw using − 80 °C EtOH and 37 °C water baths. Vero cells at a density of 1 × 10^6^ cells/well in 6-well cell culture plates (Corning Inc.; Corning, NY, USA) were infected with 500 μL of freeze-thawed cell culture supernatant. Six days post infection, when maximal GFP signal and syncytia were observed, supernatant was clarified by centrifugation (2400 rpm) and 300 μL of virus supernatant was passaged to fresh 1 × 10^6^ Vero cells in 6-well cell culture plates. Within 24 h post infection (hpi), GFP signal and syncytia were observed. After 3 days, maximal GFP signal was observed and supernatant was again clarified then passaged to fresh Vero cells (75 cm^2^ flasks) for amplification of rCedPV-GFP. Virus supernatant was concentrated by ultracentrifugation (28,000 rpm; 2 h) through a sucrose cushioned buffer. Stock rCedPV-GFP was serially diluted and incubated with Vero cells for 72 h to determine titer. Similarly, rCedPV-wt was harvested when maximal syncytia was observed, clarified and passaged onto fresh Vero cells for amplification. Stock titers of rCedPV-wt were determined by CPE-based plaque assay (see below).

### Replication kinetics of recombinant CedPV

Vero cells at a density of 5 × 10^4^ cells/well in 96-well cell culture plates were infected at a multiplicity of infection (MOI) of 1.0. Supernatants were collected at 0, 8, 24, 48 and 72 hpi and viral titers were determined by plaque assay as described by Weingartl et al. [68]. Briefly, 400 μl/well of virus inoculum was added to Vero cells at a density of 5 × 10^5^ cells/well in 12-well cell culture plates and incubated for 1 h at 37 °C, 5% CO_2_. Two mL of 2% carboxymethylcellulose sodium salt (medium viscosity) + DMEM-3% CCS was then added to each well and incubated for 5 days at 37 °C, 5% CO_2_. The plates were fixed with 4% formaldehyde and then stained with 0.5% crystal violet-80% methanol in phosphate buffered saline (PBS). Plaques were counted and calculated as PFU/mL.

### Quantitative reverse transcriptase PCR

HeLa-CCL2 cells were seeded at a density of 1.25 × 10^5^ cells/well in a 24-well plate and incubated overnight. Cells were untransfected (mock), transfected with Poly I:C (1 μg/mL) using Lipofectamine 2000 (Thermo Fisher Scientific; MA, USA), or infected with rCedPV-wt (MOI: 0.5, 1.0, 5.0). At 24 hpi, total RNA was extracted using the RNeasy Mini Kit, (Qiagen; MD, USA). An amount of 500 ng of DNase I digested RNA was converted to cDNA using the Superscript III First Strand Synthesis System for RT-PCR (Thermo Fisher Scientific) and oligo(dT) primers. Quantitative reverse transcriptase PCR (q-RT-PCR) was performed using the Power SYBR Green PCR Master Mix (Thermo Fisher Scientific) and the Applied Biosystems 7500 Real-Time PCR System. PCR cycling conditions were: 95 °C, 10 min; 40× cycles of 95 °C, 15 s; 60 °C, 1 min; with a melt curve analysis at the end of each assay. Each sample was analyzed for IFN-α, IFN-β and 18S ribosomal RNA in triplicate and fold changes were calculated relative to 18S ribosomal RNA and normalized to mock samples using the ΔΔCt method. Primer sequences are available upon request.

### Ephrin ligand-mediated fusion

HeLa-USU, HeLa-USU-EFNB2, and HeLa-USU-EFNB3 cell lines were seeded at 5 × 10^5^ cells/well in 6-well cell culture plates and incubated overnight. The next day, cells were washed with 1× PBS and received fresh cell culture medium with no virus (uninfected) or were inoculated with fresh cell culture media containing rCedPV-GFP or rCedPV-wt at an MOI: 0.1. rCedPV-GFP infected cell cultures were monitored for fluorescence and syncytia and rCedPV-wt infected cells were monitored for syncytia. At 24 and 72 hpi, all cells were fixed with methanol and stained with 0.5% crystal violet-25% methanol. Images were captured with a Zeiss Axio Observer A1 inverted microscope using the 5× objective.

### Split-luciferase based cell-cell fusion assay

The quantitative fusion assay was based on a dual-split-reporter assay [69]. HeLa-USU cells (1 × 10^4^ in a clear bottom, black wall 96-well plate) were transfected with 60 ng of the expression plasmid for the indicated receptor and 50 ng of the expression plasmid for one half of a split-luciferase reporter protein (DSP1–7, a kind gift of Z. Matsuda). As a control, HeLa-USU cells were only transfected with DSP1–7. Concurrently, HeLa-USU cells (7 × 10^5^ in a 6-well plate) were transfected with 500 ng of the other dual-split-reporter expression plasmid (DSP8–11) and 12 h later infected with rCedPV-GFP (MOI: 1.0). As an additional control, HeLa-USU cells were also mock infected. Twenty-four hours post-infection, Versene (0.48 mM EDTA in PBS) (Thermo Fisher Scientific) was used to gently detach the infected HeLa cells from the 6-well plate and 2 × 10^4^ cells overlaid on the receptor-expressing HeLa-USU cells in the 96-well plate. EnduRen (Promega; WI, USA) was added as the substrate to the culture medium (DMEM, 10% FBS) according to the manufacturer’s instructions. Content mixing between HeLa-USU cells as a result of fusion driven by interactions between virus infected cells and receptor bearing cells was monitored at the indicated times using an Infinite M200 Pro microplate reader (Tecan; Switzerland).

### Monoclonal antibody m14F3 neutralization assay

Murine monoclonal antibody (mAb) m14F3, is a CedPV soluble G specific mAb of IgG2 subclass that was developed using standard hybridoma generation techniques by immunization of BalbC mice with purified, recombinant soluble CedPV G glycoprotein produced from a human 293F stably-expressing cell line. HeLa-USU cells were seeded at a density of 5 × 10^4^ cells/well in 96-well cell culture plates. The next day, the indicated dilutions of mAb m14F3 were incubated with equal volumes of rCedPV-GFP (MOI: 0.1) for 1 h at 37 °C. was removed from the cells and 100 μl of virus or the antibody-virus mixture was added to the wells in triplicate and allowed to incubate for 1 h at 37 °C. After the incubation, the antibody-virus mixture and virus only was removed and all cells were washed once with DMEM-10. Fresh DMEM-10 with the varying concentrations of m14F3 were added to the corresponding wells and incubated for 48 h at 37 °C. GFP foci were counted with a Zeiss Axio Observer A1 inverted microscope using the 5X objective. The percent neutralization was determined based on the presence of GFP foci in replicate wells at each antibody concentration and calculated based on the reduction in the average number of foci per well to the average number of foci observed in the no antibody control, multiplied by 100 [70]**.**

### Statistical analyses

Unless otherwise stated, graphs and images are the average of two independent experiments. All experimental results were expressed as the arithmetic mean. Standard deviations were calculated and represented thusly. All statistical analyses were performed with the unpaired, two-tailed Student T-test using GraphPad’s - QuickCalcs software (GraphPad Software Inc.; CA, USA).

## Results

### Rescue of recombinant viruses

**Fig. 1:**
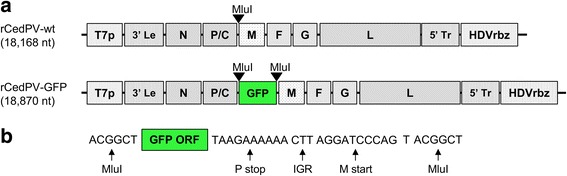
Schematic representation of the recombinant viral genomes. **a** Cloned rCedPV DNA fragments were inserted between a T7 RNA promoter and a hepatitis delta virus ribozyme in the pOLTV5 vector to yield rCedPV-wt. A MluI restriction site was created between the P and M genes to facilitate insertion of a modified turbo Green Fluorescent Protein (GFP) gene to generate rCedPV-GFP. **b** The 3′ terminus of the GFP gene is flanked by the P gene transcriptional stop, intergenic region (IGR), M gene transcriptional stop sequences. A non-coding thymine (T) between the M gene transcriptional stop and MluI sequence was added to preserve the rule of six necessary for replication

**Fig. 2:**
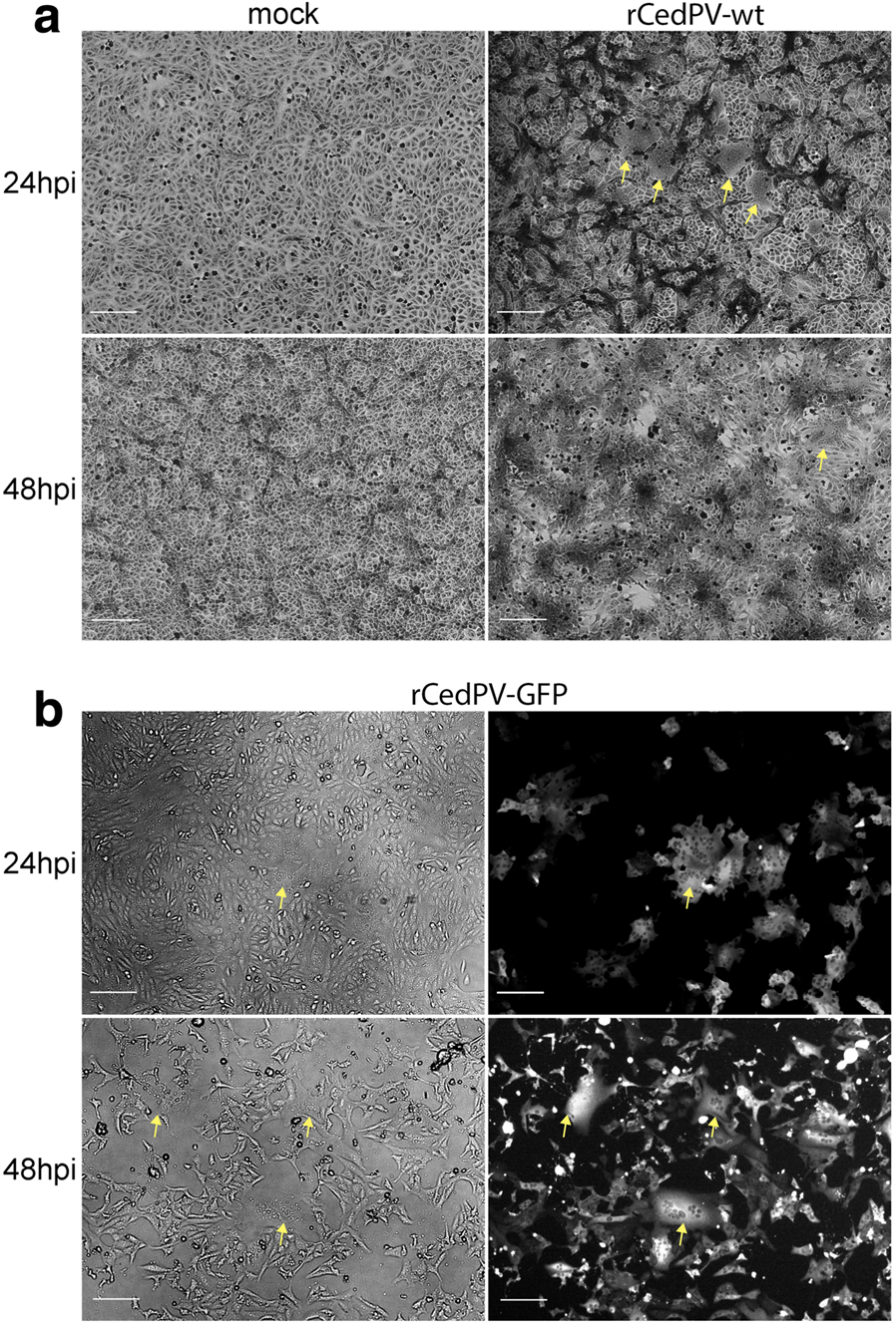
Recombinant CedPV forms syncytia in Vero cells. Vero cells were infected or not (mock) with rCedPV-wt (**a**) and rCedPV-GFP (**b**) from successful virus rescue wells. At 24 and 48 hpi, rCedPV infected cells were fixed with methanol and stained with 0.5% crystal violet-25% methanol. Images were captured with a Zeiss Axio Observer A1 inverted microscope using a 5X objective. Yellow arrows indicate giant cells and scale bar is 50 μm

A reverse genetics approach was utilized to produce rCedPV. Two rCedPV antigenome clones were created and used for virus rescue (Fig. 1a, b). The first is a recombinant wild-type CedPV (rCedPV-wt) and the second is a reporter virus that contains a turboGFP gene inserted between CedPV P and M genes (rCedPV-GFP). Observation of syncytia in Vero cells infected with cell supernatant passaged from BSR-T7/5 cells transfected with rCedPV antigenome and helper plasmids indicated successful rescue of rCedPV-wt had occurred (Fig. 2a). Rescue of rCedPV-GFP reporter was confirmed by the detection of fluorescent positive syncytia in a monolayer of Vero cells (Fig. 2b).

**Fig. 3:**
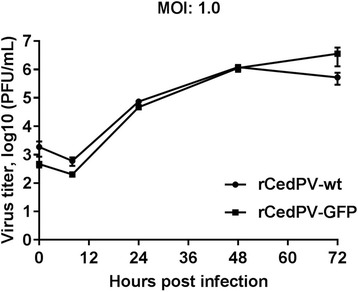
Replication kinetics of recombinant CedPV in Vero cells. Vero cells at a density of 1 × 10^6^ cells/well in 12-well cell culture plates were infected at an MOI of 1.0. Supernatants were collected at 0, 8, 24, 48 and 72 hpi and viral titers were determined by plaque assay and calculated as PFU/mL. The graphs are representative of two independent experiments performed in triplicate. Error bars indicate standard deviation

The replication kinetics of rCedPV in Vero cells infected at a MOI of 1.0 were compared (Fig. 3). At all time points examined, no statistically significant differences between rCedPV-wt and rCedPV-GFP were observed. Although there is approximately a 1 log difference between the viruses at 72 hpi, this was found not to be statistically significant with a *p*-value of 0.06. This data indicates that introduction of the reporter gene did not interfere with the growth kinetics of these recombinant viruses. Maximal titers of ~ 1-2 × 10^6^ PFU/mL were recorded between 48 hpi and 72 hpi.

### Recombinant CedPV induces an interferon response

**Fig. 4:**
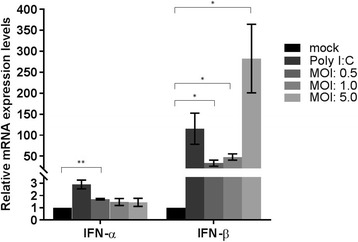
Recombinant CedPV induces an IFN response. HeLa-CCL2 cells were infected with medium with no virus (mock), infected with rCedPV-wt (MOI: 0.5, 1.0, 5.0) or transfected with Poly I:C (1 μg/mL). Twenty-four hours post-infection, total RNA was extracted, of which 500 ng was used for q-RT-PCR for IFN-α, IFN-β and 18S ribosomal RNA. Each sample was analyzed in triplicate and fold changes were calculated relative to 18S ribosomal RNA and normalized to mock samples using the ΔΔCt method. The graphs are representative of two independent experiments performed in triplicate. Error bars indicate standard deviation.* *p* < 0.05 and ** *p* < 0.01

Transfection experiments with CedPV P protein demonstrated that the IFN response in HEK293T or HeLa-CCL2 cells was less antagonized when to compared to IFN signaling antagonism with HeV P protein [50] and infection with wild-type CedPV stimulated the IFN-β response in infected HeLa-CCL2 cells [49]. To further characterize the phenotype of our rCedPV, we examined the type I IFN response in rCedPV infected human cells. Poly I:C served as a positive control to demonstrate that the IFN production pathway was functional in the HeLa-CCL2 cells. Total RNA was extracted from uninfected (mock), Poly I:C treated and rCedPV-wt infected HeLa-CCL2 cells at 24 hpi and IFN-α and IFN-β mRNA levels were quantified by q-RT-PCR. As seen in Fig. 4, in contrast to the mock infected samples, rCedPV-wt induced a robust IFN-β response ranging from 33 to 283 fold increase, in a dose-dependent manner. The increase in IFN-β mRNA levels of rCedPV-wt samples was statistically significant at all MOIs tested when compared to the mock infected samples (*p* < 0.05). In addition, we observed a 1.5–1.7 fold increase of IFN-α in rCedPV-wt infected samples when compared to the mock infected samples, which was statistically significant only at the lowest MOI of 0.5 (*p* < 0.01). This data demonstrates that at 24 hpi, analogous to the wild-type CedPV [49], rCedPV induced a robust IFN-β response, while maintaining IFN-α levels similar to that observed in the mock infected samples. The precise mechanism of the Type-I IFN response in rCedPV infected cells warrants further investigation and is currently under study.

### Ephrin receptor tropism of recombinant CedPV

**Fig. 5:**
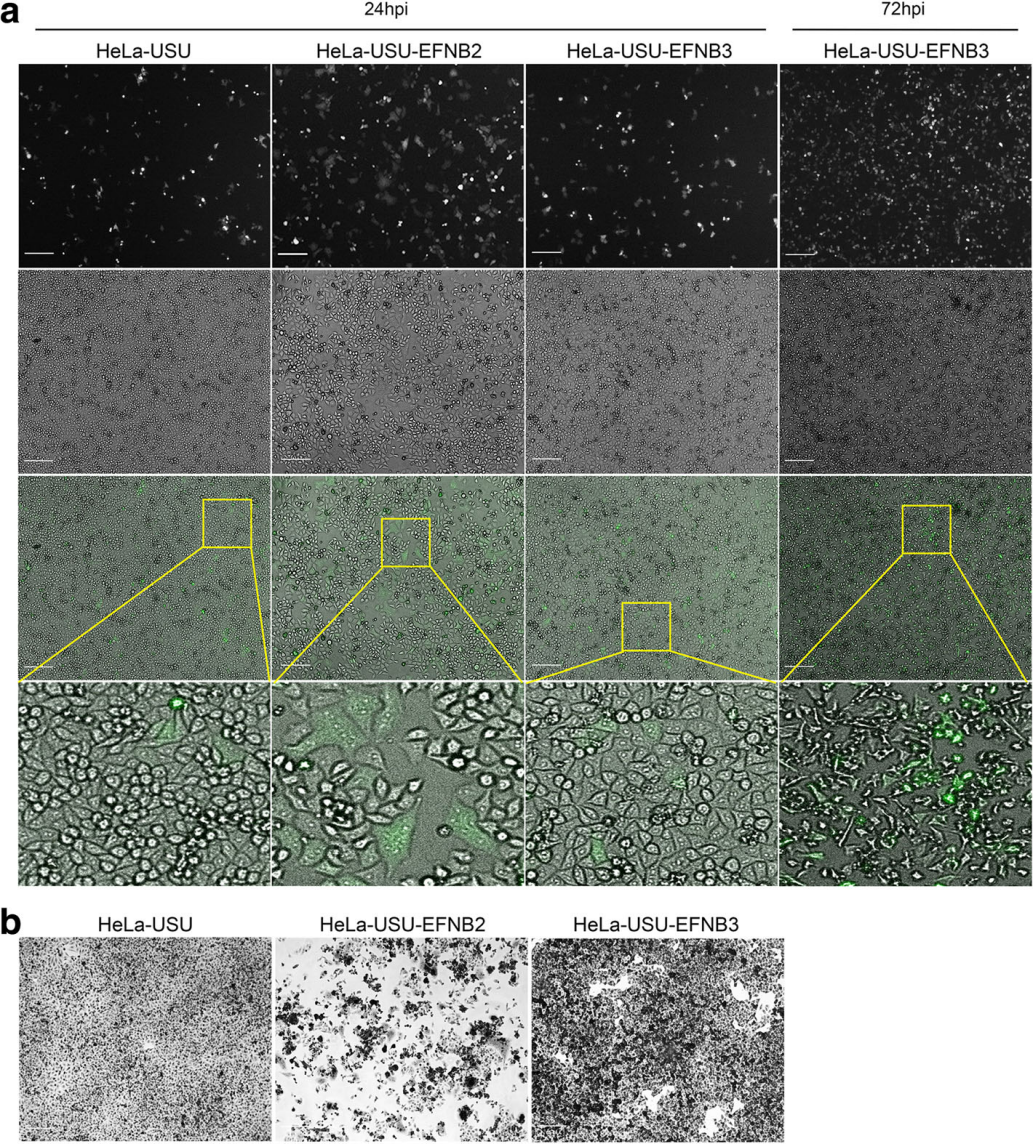
Ephrin B2 is recognized as an entry receptor by rCedPV. HeLa-USU, HeLa-USU-EFNB2, and HeLa-USU-EFNB3 cells at a density of 1 × 10^6^ cells/well in a 6-well cell culture plates were infected with medium with no virus (uninfected) or with medium containing rCedPV-GFP (**a**) or rCedPV-wt (**b**) at an MOI of 0.1. **a** rCedPV-GFP infected cell cultures were monitored for fluorescence and syncytia by microscopy at 24 and 72 hpi; HeLa-USU-EFNB3 cells 72 hpi are shown. **b** rCedPV-wt infected cells were monitored for syncytia at 24 and 72 hpi; the latter is shown. Cells were fixed with methanol and stained with 0.5% crystal violet-25% methanol. Images were captured with a Zeiss Axio Observer A1 inverted microscope using a 5X objective. Scale bar at 50 μm and insets show zoom magnified areas of the cell monolayer

Membrane-bound EFNB2 and EFNB3 facilitate the entry of HeV and NiV into host cells [16–18]. Characterization of wild-type CedPV demonstrated that receptor triggered cell fusion was mediated by EFNB2, but not EFNB3 [49]. The HeLa-USU cell line has been used as a henipavirus entry negative cell line and artificial expression of EFNB2 in the HeLa-USU receptor negative background was used to identify EFNB2 as the cellular receptor of both HeV and NiV, and the wild-type CedPV isolate [16, 49, 71]. To assess whether our rCedPV has similar receptor tropism to the wild-type CedPV isolate, HeLa-USU cells and HeLa-USU cells stably expressing either EFNB2 or EFNB3 were infected with rCedPV-GFP and rCedPV-wt. Unexpectedly, GFP expression was observed in all three cell lines: HeLa-USU, HeLa-USU-EFNB2, and HeLa-USU-EFNB3 (Fig. 5a). However, syncytia following rCedPV-GFP was observed only in HeLa-USU-EFNB2 cells as early as 24 hpi, but not in HeLa-USU-EFNB3 cells at 24 hpi or 72 hpi, which had no observable syncytia and little cytopathogenic effects (CPE) at the later time point. Similarly, the rCedPV-wt infected HeLa-USU exhibited extensive syncytial CPE at 72 hpi when EFNB2 was expressed, and this phenotype was not observed with HeLa-USU cells or HeLa-USU-EFNB3, which remained as healthy monolayers at this post-infection time point (Fig. 5b). Although GFP expression was observed in both HeLa-USU and HeLa-USU-EFNB3 cells infected with rCedPV-GFP, syncytia was not observed and GFP expression appeared to indicate single cell infection. These results further supported the requirement for EFNB2 as a receptor for virus entry and syncytia formation, but also highlighted an uncharacterized, unknown mechanism that facilitated low levels of rCedPV entry that do not result in syncytia and which, without the presence of a reporter gene would have gone unnoticed.

**Fig. 6:**
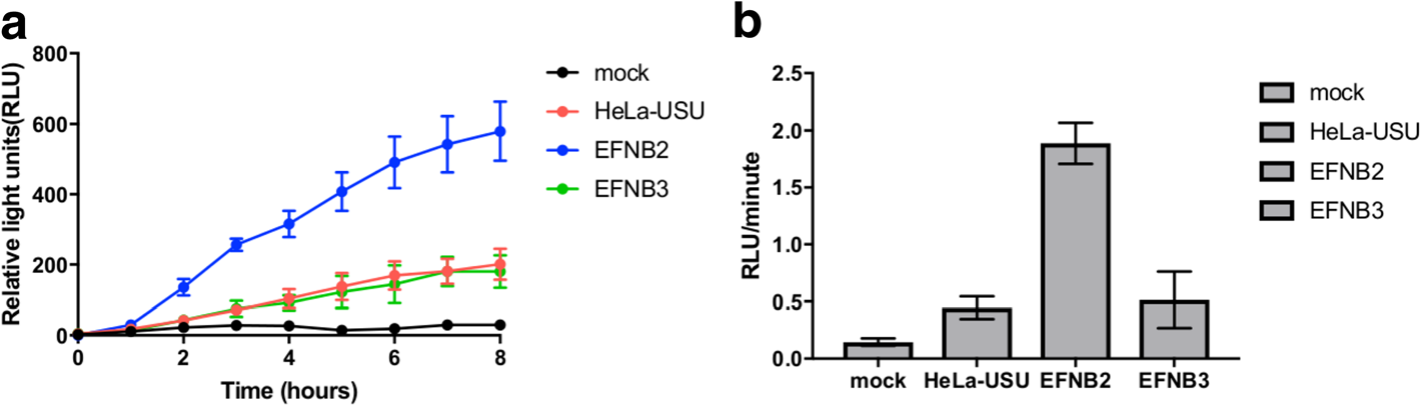
Cell-cell fusion kinetics of HeLa-USU cells mediated by rCedPV. **a** HeLa-USU cells in a 96-well plate were transfected with the indicated receptor and a plasmid encoding one half of a split-luciferase reporter protein. Concurrently HeLa-USU cells in a 6-well were infected or not (mock) with rCedPV-GFP (MOI 1.0) and transfected with the other half of the split-luciferase. Thirty-six hours post infection the cells were re-suspended and overlaid on the receptor expressing HeLa-USU cells in the 96-well plate. The live-cell luciferase substrate EnduRen was used to monitor the level of cell-cell fusion at the indicated time points. **b** The rate of fusion was calculated between hours 1 and 3 as the slope of the curve. The graphs are representative of two independent experiments performed in technical duplicates. Error bars indicate standard deviation

Next, we used a quantitative split-luciferase based reporter assay [69] to compare levels of fusion in standard and EFNB2-positive HeLa-USU cells. In this assay, content mixing between virus-infected effector cells expressing the rCedPV fusion complex and a target cell expressing or not the known henipavirus receptors allows the two halves of *Renilla* luciferase to functionally interact. As expected, HeLa-USU cells transiently expressing EFNB2 exhibited the highest levels and fastest kinetics of cell-cell fusion (Fig. 6a). On the other hand, the standard HeLa-USU cells also supported cell-cell fusion, although to a 3× lower level and at a 4× slower rate (Fig. 6a, b, respectively). The over-expression of EFNB3 did not increase the level or rate of fusion over that observed for the standard HeLa-USU cells, suggesting that CedPV cannot use this ligand for cell entry. Taken together, the data presented in Figs. 5 and 6 indicated that rCedPV utilized EFNB2 for cellular entry and fusion.

### Neutralization of recombinant CedPV-GFP infection

**Fig. 7:**
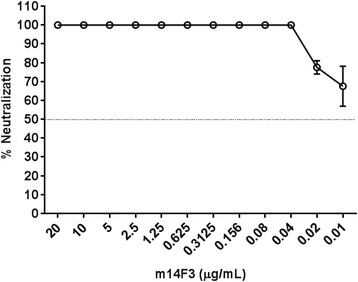
rCedPV-GFP entry into HeLa-USU cells is inhibited by m14F3. m14F3 was incubated with equal volume of rCedPV-GFP (MOI: 0.1) for 1 h at 37 °C and the mixture was added to HeLa-USU cells. After a 1 h incubation at 37 °C, antibody-virus mixtures were removed, cells were washed, and medium containing fresh antibody was added to the cells and incubated for an additional 48 h. The graph is the average of two independent experiments performed in triplicate. Error bars indicate standard deviation

To determine whether the apparent low level of rCedPV infection in HeLa-USU cells observed by GFP reporter signal was CedPV envelope glycoprotein specific entry, we performed a virus neutralization assay targeting the CedPV G attachment glycoprotein with the neutralizing mAb, m14F3. Incubation of rCedPV-GFP with m14F3 completely (100%) neutralized rCedPV-GFP at 10 of the 12 m14F3 concentrations tested. The two lowest concentrations of 0.02 and 0.01 μg/mL decreased rCedPV-GFP entry by 77% and 67% respectively (Fig. 7). These results demonstrated that the low level of rCedPV infection of HeLa-USU cells as detected by the presence of GFP reporter signal is mediated by the G glycoprotein.

## Discussion

In this study, we used a reverse genetics approach to rescue rCedPV, and characterized the in vitro functionality of rCedPV. In the first report of CedPV isolation and characterization, Marsh et al. demonstrated that CedPV lacks the coding capacity for the IFN antagonist V protein and that CedPV infection of HeLa-CCL2 cells stimulates the production of IFN-β [49]. Although we cannot directly compare CedPV and rCedPV because the original isolate remains in a secure laboratory, we demonstrate that the rCedPV rescued in this study also stimulates the production of IFN-β. The HeV and NiV P gene encodes accessory proteins, V and W, are potent antagonists of the toll-like receptor signaling and IFN pathways, by sequestering and inhibiting phosphorylation of STAT1 and STAT2 [51–56]. This suppression of a type I IFN response may contribute to the high virulence and pathogenicity observed with these viruses [57, 62]. In contrast, the CedPV P gene does not undergo RNA editing and therefore does not encode for V or W proteins [49, 50]. Furthermore, Lieu et al., performed a sequence alignment of the residues in the P protein that are essential for STAT1 binding and showed that CedPV-P shares only 13% sequence identity with NiV-P and HeV-P in those specified residues and therefore should not interact with STAT1 [50]. Interestingly, in CedPV infected HeLa cells IFN-α mediated STAT1 nuclear trafficking was slightly impaired; however, not to the extent inhibited by HeV [50]. This suggested that CedPV may encode viral factors, such as the C protein produced from an alternate reading frame that may be able to interfere with STAT1 nuclear translocation [50]. In fact, NiV C protein weakly inhibited IFN signaling but the mechanism has yet to be elucidated [62, 72]. Henipaviruses have multiple strategies to antagonize the IFN response and therefore to determine the precise relationship between virulence and the inhibition of IFN requires studies with mutant-virus in susceptible animal models.

We confirmed that rCedPV utilizes the HeV and NiV receptor EFNB2 for entry into human cells. Unexpectedly, we observed that rCedPV-GFP infected standard HeLa-USU cells, which are EFNB2-receptor-negative, expressed GFP, indicative of rCedPV entry and replication. However, syncytia were only observed in HeLa-USU-EFNB2, but not HeLa-USU or HeLa-USU-EFNB3 cells (Fig. 5a, b). Entry of rCedPV into an EFNB2 negative cell line, HeLa-USU, was corroborated by analysis of virus mediated cell-cell fusion kinetics (Fig. 6a, b), which showed a higher background level of cell-cell fusion than what would be expected for a receptor negative cell line.

Transcriptomic analysis of our parental HeLa-USU cell line demonstrated that this cell line was negative for EFN-B type ligands [16]. Using the formation of giant cells as a marker for CedPV infection, Marsh et al. identified EFNB2 as the receptor for CedPV in the HeLa-USU cell line expressing EFNB2; however, entry into HeLa-USU cells was not noted by this group as only syncytia formation was used as read out of entry [49]. We also did not observe CPE or syncytia in HeLa-USU or HeLa-USU-EFNB3 cell lines, and infection of these cells would not have been noticed if not for the GFP reporter gene (Fig. 5a). In our study, GFP expression in HeLa-USU cells infected with rCedPV-GFP is suggestive that endogenous expression of an uncharacterized cellular receptor is facilitating rCedPV entry. Viral envelope glycoprotein mediated receptor binding and fusion activation facilitates cellular entry of henipaviruses (reviewed in: [73]) and we demonstrated here that rCedPV entry could be completely blocked by a CedPV G specific mAb. Cell to cell fusion is dependent on both the amount of virus receptor and virus envelope glycoproteins (F and G) present [74, 75], which is perhaps why in the presence of an uncharacterized receptor that facilitates either a) low level of entry as seen by GFP expression in single cells or b) inefficient receptor-mediated fusion that does not support giant cell formation. However, at this time we are not able to experimentally rule out the possibility that despite previous transcriptomic analysis of our HeLa-USU cell line, a low-level of EFNB2 expression currently persists and is facilitating the presently observed low-levels of rCedPV-GFP entry.

HeV and NiV do not utilize glycosylphosphatidylinositol-linked EFN-A type ligands or EFNB1 for cellular entry [19], and studies of cellular receptors for related henipaviruses, KumPV and MojPV, have revealed some similarities and differences in EFNB2 and EFNB3 recognition. The African henipavirus, KumPV, G glycoprotein mediated receptor binding and triggered fusion with EFNB2 [76, 77]. Further analysis confirmed that KumPV G binds to EFNB2, but not EFNB3, and structural analysis demonstrated that despite a low sequence identity between the G glycoprotein of NiV and KumPV, the amino acid residues in the globular domain that bind to EFNB2 are highly conserved [78]. The cellular receptor of MojPV remains elusive, but MojPV does not bind to EFNB2 or EFNB3, and is the first *Henipavirus* species with this described functional divergence in receptor utility [79]. Despite low sequence identity among the G proteins of CedPV, HeV, NiV, and KumPV, these henipaviruses have highly conserved amino acids in the receptor binding domain of G [22]. Future functional analysis of CedPV F and G and rCedPV tropism will allow us to further investigate the nature of the cellular receptors that are facilitating rCedPV entry into HeLa-USU cells.

In addition to the observed novel receptor tropism, rCedPV can be used as a platform to explore new henipavirus therapeutics. The experimental vaccines and therapeutics currently available for HeV and NiV infection are based on the virus envelope glycoproteins; however, these therapeutics are not presently licensed for human use [80–82]. A HeV G glycoprotein subunit vaccine, Equivac® HeV, which breaks the chain of zoonotic transmission from bats to horses to humans, is licensed for horse vaccination in Australia and is also a promising human vaccine antigen candidate that is protective against HeV or NiV challenge in ferret and non-human primate infection models [83–86]. A human monoclonal antibody (hmAb), m102.4, is cross-protective against HeV and NiV challenge in the ferret and non-human primate models [87–90] and has been administered by compassionate use to people after natural or lab-based exposure to HeV and lab-based exposure to NiV [81]. Structural studies of the HeV and NiV G glycoprotein in complex with m102.4 revealed that the hmAb recognizes the receptor-binding domain in the G glycoprotein blocking its interaction with EFN receptors [91].

Serological evidence of henipavirus infections has been detected in both bats and humans in Cameroon, suggesting zoonotic transmission of African henipaviruses [45]. The population of *Eidolon helvum*, a bat natural host of African henipaviruses [43–45, 92] ranges widely throughout Sub-Saharan Africa, which raises concerns about the potential of zoonotic transmission [92]. Whether therapeutics that are cross-protective against HeV and NiV will be efficacious against African henipaviruses is unknown. Polyclonal serum raised against NiV was demonstrated to less potently cross-neutralize African henipavirus, KumPV [45]. This finding highlighted the possibility that therapeutics based on the G glycoprotein of HeV and NiV might not be effective against less antigenically-related emerging African or novel Asiatic henipaviruses such as KumPV or MojPV. We seek to use rCedPV as a vaccine platform to explore novel pan-henipavirus therapeutics that target either viral proteins more conserved than G or henipavirus life-cycle stages. This strategy has the potential to address emerging henipaviruses, a shortcoming of present therapeutic approaches.

## Conclusions

CedPV is the third *Henipavirus* species to have been isolated from *Pteropus* fruit bats, and is presently the only non-pathogenic *Henipavirus* species identified and characterized. Here, we describe the successful rescue of two recombinant CedPV variants using a reverse genetics approach. As the wild-type CedPV isolate, our recombinant viruses induce an IFN-β response in infected human cells and as expected, receptor mediated fusion was triggered by EFNB2 but not EFNB3. Our rCedPV platform can be used to study various aspects of henipavirus cell biology and host cell interactions, as well as an authentic henipavirus platform for antiviral drug discovery or vaccine approaches safely under BSL-2 laboratory containment.

## Abbreviations

BSL: Biological safety level
CedPV: Cedar virus
CPE: Cytopathogenic effects
DSP1–7/DSP8–11: Dual split-luciferase reporter proteins
EFN: Ephrin ligand
EFNB2: Ephrin-B2 ligand
EFNB3: Ephrin-B3 ligand
F: Fusion glycoprotein
G: Attachment glycoprotein
GFP: Green fluorescent protein
HDV: Hepatitis delta virus
HeV: Hendra virus
hmAb: Human monoclonal antibody
IFN: Interferon
KumPV: Kumasi virus
MOI: Multiplicity of infection
MojPV: *Mojiang henipavirus*
NiV: Nipah virus
pCMV: Cytomegalovirus promoter
q-RT-PCR: Quantitative reverse transcriptase polymerase chain reaction
rCedPV: Recombinant cedar virus
wt: Wild-type

## References

[1] Wang LF, Yu M, Hansson E, Pritchard LI, Shiell B, Michalski WP (2000). The exceptionally large genome of Hendra virus: support for creation of a new genus within the family Paramyxoviridae. J Virol..

[2] Clayton BA (2017). Nipah virus: transmission of a zoonotic paramyxovirus. Curr Opin Virol..

[3] Luby SP, Broder CC, Le Duc JWKRSL (2014). Paramyxoviruses: Henipaviruses. Viral Infections of Humans, Epidemiology and Control.

[4] Wang LF, Mackenzie JS, Broder CC, Knipe DMHPM (2013). Henipaviruses. Fields Virology.

[5] Selvey LA, Wells RM, McCormack JG, Ansford AJ, Murray K, Rogers RJ (1995). Infection of humans and horses by a newly described morbillivirus. Med J Aust..

[6] Centers for Disease Control and Prevention (CDC). Outbreak of Hendra-like virus--Malaysia and Singapore, 1998-1999. MMWR Morb Mortal Wkly Rep. 1999;48:265–9. 10227800

[7] Paton NI, Leo YS, Zaki SR, Auchus AP, Lee KE, Ling AE (1999). Outbreak of Nipah-virus infection among abattoir workers in Singapore. Lancet..

[8] Chua KB, Goh KJ, Wong KT, Kamarulzaman A, Tan PS, Ksiazek TG (1999). Fatal encephalitis due to Nipah virus among pig-farmers in Malaysia. Lancet..

[9] Chua KB, Bellini WJ, Rota PA, Harcourt BH, Tamin A, Lam SK (2000). Nipah virus: a recently emergent deadly paramyxovirus. Science..

[10] Chua KB (2003). Nipah virus outbreak in Malaysia. J Clin Virol..

[11] Tan CT, Wong KT (2003). Nipah encephalitis outbreak in Malaysia. Ann Acad Med Singapore..

[12] Hsu VP, Hossain MJ, Parashar UD, Ali MM, Ksiazek TG, Kuzmin I (2004). Nipah virus encephalitis reemergence, Bangladesh. Emerg Infect Dis..

[13] Harcourt BH, Lowe L, Tamin A, Liu X, Bankamp B, Bowden N (2005). Genetic characterization of Nipah virus, Bangladesh, 2004. Emerg Infect Dis..

[14] Chadha MS, Comer JA, Lowe L, Rota PA, Rollin PE, Bellini WJ (2006). Nipah virus-associated encephalitis outbreak, Siliguri, India. Emerg Infect Dis.

[15] Eaton BT, Broder CC, Middleton D, Wang LF (2006). Hendra and Nipah viruses: different and dangerous. Nat Rev Microbiol..

[16] Bonaparte MI, Dimitrov AS, Bossart KN, Crameri G, Mungall BA, Bishop KA (2005). Ephrin-B2 ligand is a functional receptor for Hendra virus and Nipah virus. Proc Natl Acad Sci U S A..

[17] Negrete OA, Levroney EL, Aguilar HC, Bertolotti-Ciarlet A, Nazarian R, Tajyar S (2005). EphrinB2 is the entry receptor for Nipah virus, an emergent deadly paramyxovirus. Nature..

[18] Xu K, Broder CC, Nikolov DB (2012). Ephrin-B2 and ephrin-B3 as functional henipavirus receptors. Semin Cell Dev Biol..

[19] Negrete OA, Wolf MC, Aguilar HC, Enterlein S, Wang W, Mühlberger E (2006). Two key residues in ephrinB3 are critical for its use as an alternative receptor for nipah virus. PLoS Pathog..

[20] Bishop KA, Stantchev TS, Hickey AC, Khetawat D, Bossart KN, Krasnoperov V (2007). Identification of Hendra virus G glycoprotein residues that are critical for receptor binding. J Virol..

[21] Gale NW, Baluk P, Pan L, Kwan M, Holash J, DeChiara TM (2001). Ephrin-B2 selectively marks arterial vessels and neovascularization sites in the adult, with expression in both endothelial and smooth-muscle cells. Dev Biol..

[22] Pernet O, Wang YE, Lee B (2012). Henipavirus receptor usage and tropism. Curr Top Microbiol Immunol..

[23] Hooper P, Zaki S, Daniels P, Middleton D (2001). Comparative pathology of the diseases caused by Hendra and Nipah viruses. Microbes Infect..

[24] Wong KT, Shieh W-J, Kumar S, Norain K, Abdullah W, Guarner J (2002). Nipah virus infection: pathology and pathogenesis of an emerging paramyxoviral zoonosis. Am J Pathol..

[25] Maisner A, Neufeld J, Weingartl H (2009). Organ- and endotheliotropism of Nipah virus infections in vivo and in vitro. Thromb Haemost..

[26] Vigant F, Lee B (2011). Hendra and nipah infection: pathology, models and potential therapies. Infect Disord Drug Targets..

[27] Williamson MM, Hooper PT, Selleck PW, Gleeson LJ, Daniels PW, Westbury HA (1998). Transmission studies of Hendra virus (equine morbillivirus) in fruit bats, horses and cats. Aust Vet J..

[28] Westbury HA (2000). Hendra virus disease in horses. Rev Sci Tech..

[29] Williamson MM, Hooper PT, Selleck PW, Westbury HA, Slocombe RF (2000). Experimental hendra virus infectionin pregnant guinea-pigs and fruit Bats (*Pteropus poliocephalus*). J Comp Pathol..

[30] Middleton DJ, Westbury HA, Morrissy CJ, van der Heide BM, Russell GM, Braun MA (2002). Experimental Nipah virus infection in pigs and cats. J Comp Pathol..

[31] Wong KT, Grosjean I, Brisson C, Blanquier B, Fevre-Montange M, Bernard A (2003). A golden hamster model for human acute Nipah virus infection. Am J Pathol..

[32] Negrete OA, Chu D, Aguilar HC, Lee B (2007). Single amino acid changes in the Nipah and Hendra virus attachment glycoproteins distinguish ephrinB2 from ephrinB3 usage. J Virol..

[33] Halpin K, Young PL, Field H, Mackenzie JS (1999). Newly discovered viruses of flying foxes. Vet Microbiol..

[34] Halpin K, Young PL, Field HE, Mackenzie JS (2000). Isolation of Hendra virus from pteropid bats: a natural reservoir of Hendra virus. J Gen Virol..

[35] Chua KB, Koh CL, Hooi PS, Wee KF, Khong JH, Chua BH (2002). Isolation of Nipah virus from Malaysian Island flying-foxes. Microbes Infect..

[36] Halpin K, Hyatt AD, Fogarty R, Middleton D, Bingham J, Epstein JH (2011). Pteropid bats are confirmed as the reservoir hosts of henipaviruses: a comprehensive experimental study of virus transmission. Am J Trop Med Hyg..

[37] Epstein JH, Field HE, Luby S, Pulliam JR, Daszak P (2006). Nipah virus: impact, origins, and causes of emergence. Curr Infect Dis Rep..

[38] Plowright RK, Eby P, Hudson PJ, Smith IL, Westcott D, Bryden WL (2015). Ecological dynamics of emerging bat virus spillover. Proc Biol Sci..

[39] Olson JG, Rupprecht C, Rollin PE, An US, Niezgoda M, Clemins T (2002). Antibodies to Nipah-like virus in bats (*Pteropus lylei*), Cambodia. Emerg Infect Dis..

[40] Wacharapluesadee S, Boongird K, Wanghongsa S, Ratanasetyuth N, Supavonwong P, Saengsen D (2010). A longitudinal study of the prevalence of Nipah virus in Pteropus lylei bats in Thailand: evidence for seasonal preference in disease transmission. Vector-Borne and Zoonotic Diseases..

[41] Sendow I, Ratnawati A, Taylor T, Adjid RMA, Saepulloh M, Barr J (2013). Nipah virus in the fruit bat *Pteropus vampyrus* in Sumatera, Indonesia. PLoS One..

[42] Iehle C, Razafitrimo G, Razainirina J, Andriaholinirina N, Goodman SM, Faure C (2007). Henipavirus and Tioman virus antibodies in pteropodid bats. Madagascar. Emerg Infect Dis..

[43] Hayman DT, Suu-Ire R, Breed AC, McEachern JA, Wang L, Wood JL (2008). Evidence of henipavirus infection in West African fruit bats. PLoS One..

[44] Peel AJ, Baker KS, Crameri G, Barr JA, Hayman DT, Wright E (2012). Henipavirus neutralising antibodies in an isolated island population of African fruit bats. PLoS One..

[45] Pernet O, Schneider BS, Beaty SM, LeBreton M, Yun TE, Park A (2014). Evidence for henipavirus spillover into human populations in Africa. Nat Commun..

[46] Drexler JF, Corman VM, Gloza-Rausch F, Seebens A, Annan A, Ipsen A (2009). Henipavirus RNA in African bats. PLoS One..

[47] Drexler JF, Corman VM, Muller MA, Maganga GD, Vallo P, Binger T (2012). Bats host major mammalian paramyxoviruses. Nat Commun..

[48] Wu Z, Yang L, Yang F, Ren X, Jiang J, Dong J (2014). Novel Henipa-like virus, Mojiang Paramyxovirus, in rats, China, 2012. Emerg Infect Dis..

[49] Marsh GA, de Jong C, Barr JA, Tachedjian M, Smith C, Middleton D (2012). Cedar virus: a novel Henipavirus isolated from Australian bats. PLoS Pathog..

[50] Lieu KG, Marsh GA, Wang L-F, Netter HJ (2015). The non-pathogenic Henipavirus Cedar paramyxovirus phosphoprotein has a compromised ability to target STAT1 and STAT2. Antiviral Res..

[51] Shaw ML, Garcia-Sastre A, Palese P, Basler CF (2004). Nipah virus V and W proteins have a common STAT1-binding domain yet inhibit STAT1 activation from the cytoplasmic and nuclear compartments, respectively. J Virol..

[52] Shaw ML, Cardenas WB, Zamarin D, Palese P, Basler CF (2005). Nuclear localization of the Nipah virus W protein allows for inhibition of both virus- and toll-like receptor 3-triggered signaling pathways. J Virol..

[53] Ciancanelli MJ, Volchkova VA, Shaw ML, Volchkov VE, Basler CF (2009). Nipah virus sequesters inactive STAT1 in the nucleus via a P gene-encoded mechanism. J Virol..

[54] Rodriguez JJ, Wang LF, Horvath CM (2003). Hendra virus V protein inhibits interferon signaling by preventing STAT1 and STAT2 nuclear accumulation. J Virol..

[55] Shaw ML (2009). Henipaviruses employ a multifaceted approach to evade the antiviral interferon response. Viruses..

[56] Rodriguez JJ, Horvath CM (2004). Host evasion by emerging paramyxoviruses: Hendra virus and Nipah virus v proteins inhibit interferon signaling. Viral Immunol..

[57] Satterfield BA, Cross RW, Fenton KA, Agans KN, Basler CF, Geisbert TW (2015). The immunomodulating V and W proteins of Nipah virus determine disease course. Nat Commun..

[58] Palese P, Zheng H, Engelhardt OG, Pleschka S, García-Sastre A (1996). Negative-strand RNA viruses: genetic engineering and applications. Proc Natl Acad Sci U S A..

[59] Pfaller CK, Cattaneo R, Schnell MJ (2015). Reverse genetics of Mononegavirales: How they work, new vaccines, and new cancer therapeutics. Virology.

[60] Yoneda M, Guillaume V, Ikeda F, Sakuma Y, Sato H, Wild TF (2006). Establishment of a Nipah virus rescue system. Proc Natl Acad Sci U S A..

[61] Freiberg A, Dolores LK, Enterlein S, Flick R (2008). Establishment and characterization of plasmid-driven minigenome rescue systems for Nipah virus: RNA polymerase I- and T7-catalyzed generation of functional paramyxoviral RNA. Virology..

[62] Yoneda M, Guillaume V, Sato H, Fujita K, Georges-Courbot M-C, Ikeda F (2010). The nonstructural proteins of Nipah virus play a key role in pathogenicity in experimentally infected animals. PLoS One..

[63] Marsh GA, Virtue ER, Smith I, Todd S, Arkinstall R, Frazer L (2013). Recombinant Hendra viruses expressing a reporter gene retain pathogenicity in ferrets. Virol J..

[64] Yun T, Park A, Hill TE, Pernet O, Beaty SM, Juelich TL (2015). Efficient reverse genetics reveals genetic determinants of budding and fusogenic differences between Nipah and Hendra viruses and enables real-time monitoring of viral spread in small animal models of henipavirus infection. J Virol..

[65] Peeters BP, de Leeuw OS, Koch G, Gielkens AL (1999). Rescue of Newcastle disease virus from cloned cDNA: evidence that cleavability of the fusion protein is a major determinant for virulence. J Virol..

[66] Shagin DA, Barsova EV, Yanushevich YG, Fradkov AF, Lukyanov KA, Labas YA (2004). GFP-like proteins as ubiquitous metazoan superfamily: evolution of functional features and structural complexity. Mol Biol Evol..

[67] Chan YP, Yan L, Feng YR, Broder CC (2009). Preparation of recombinant viral glycoproteins for novel and therapeutic antibody discovery. Methods Mol Biol..

[68] Weingartl HM, Berhane Y, Caswell JL, Loosmore S, Audonnet J-C, Roth JA (2006). Recombinant nipah virus vaccines protect pigs against challenge. J Virol.

[69] Navaratnarajah CK, Rosemarie Q, Cattaneo R (2015). A structurally unresolved head segment of defined length favors proper measles virus hemagglutinin tetramerization and efficient membrane fusion triggering. J Virol..

[70] Zhu Z, Dimitrov AS, Bossart KN, Crameri G, Bishop KA, Choudhry V (2006). Potent neutralization of Hendra and Nipah viruses by human monoclonal antibodies. J Virol..

[71] Bossart KN, Tachedjian M, McEachern JA, Crameri G, Zhu Z, Dimitrov DS (2008). Functional studies of host-specific ephrin-B ligands as Henipavirus receptors. Virology..

[72] Park M-S, Shaw ML, Muñoz-Jordan J, Cros JF, Nakaya T, Bouvier N (2003). Newcastle disease virus (NDV)-based assay demonstrates interferon-antagonist activity for the NDV V protein and the Nipah virus V, W, and C proteins. J Virol..

[73] Bossart KN, Fusco DL, Broder CC (2013). Paramyxovirus entry. Adv Exp Med Biol..

[74] Iorio RM, Melanson VR, Mahon PJ (2009). Glycoprotein interactions in paramyxovirus fusion. Future Virol..

[75] Chang A, Dutch RE (2012). Paramyxovirus fusion and entry: multiple paths to a common end. Viruses..

[76] Krüger N, Hoffmann M, Weis M, Drexler JF, Müller MA, Winter C (2013). Surface glycoproteins of an African Henipavirus induce syncytium formation in a cell line derived from an African fruit bat, *Hypsignathus monstrosus*. J Virol..

[77] Weis M, Behner L, Hoffmann M, Krüger N, Herrler G, Drosten C (2014). Characterization of African bat henipavirus GH-M74a glycoproteins. J Gen Virol..

[78] Lee B, Pernet O, Ahmed AA, Zeltina A, Beaty SM, Bowden TA (2015). Molecular recognition of human ephrinB2 cell surface receptor by an emergent African henipavirus. Proc Natl Acad Sci U S A..

[79] Rissanen I, Ahmed AA, Azarm K, Beaty S, Hong P, Nambulli S (2017). Idiosyncratic Mòjiāng virus attachment glycoprotein directs a host-cell entry pathway distinct from genetically related henipaviruses. Nat Commun..

[80] Broder CC, Xu K, Nikolov DB, Zhu Z, Dimitrov DS, Middleton D (2013). A treatment for and vaccine against the deadly Hendra and Nipah viruses. Antiviral Res..

[81] Broder CC, Weir DL, Reid PA (2016). Hendra virus and Nipah virus animal vaccines. Vaccine..

[82] Broder CC, Geisbert TW, Xu K, Nikolov DB, Wang L-F, Middleton D (2012). Immunization strategies against henipaviruses. Curr Top Microbiol Immunol..

[83] Middleton D, Pallister J, Klein R, Feng YR, Haining J, Arkinstall R (2014). Hendra virus vaccine, a one health approach to protecting horse, human, and environmental health. Emerg Infect Dis..

[84] Pallister J, Middleton D, Wang LF, Klein R, Haining J, Robinson R (2011). A recombinant Hendra virus G glycoprotein-based subunit vaccine protects ferrets from lethal Hendra virus challenge. Vaccine..

[85] Bossart KN, Rockx B, Feldmann F, Brining D, Scott D, LaCasse R (2012). A Hendra virus G glycoprotein subunit vaccine protects African green monkeys from Nipah virus challenge. Sci Transl Med.

[86] Mire CE, Geisbert JB, Agans KN, Feng Y-R, Fenton KA, Bossart KN (2014). A recombinant Hendra virus G glycoprotein subunit vaccine protects nonhuman primates against Hendra virus challenge. J Virol..

[87] Zhu Z, Bossart KN, Bishop KA, Crameri G, Dimitrov AS, McEachern JA (2008). Exceptionally potent cross-reactive neutralization of Nipah and Hendra viruses by a human monoclonal antibody. J Infect Dis..

[88] Bossart KN, Zhu Z, Middleton D, Klippel J, Crameri G, Bingham J (2009). A neutralizing human monoclonal antibody protects against lethal disease in a new ferret model of acute nipah virus infection. PLoS Pathog..

[89] Bossart KN, Geisbert TW, Feldmann H, Zhu Z, Feldmann F, Geisbert JB (2011). A neutralizing human monoclonal antibody protects african green monkeys from hendra virus challenge. Sci Transl Med.

[90] Geisbert TW, Mire CE, Geisbert JB, Chan YP, Agans KN, Feldmann F (2014). Therapeutic treatment of Nipah virus infection in nonhuman primates with a neutralizing human monoclonal antibody. Sci Transl Med.

[91] Xu K, Rockx B, Xie Y, DeBuysscher BL, Fusco DL, Zhu Z (2013). Crystal structure of the Hendra virus attachment G glycoprotein bound to a potent cross-reactive neutralizing human monoclonal antibody. PLoS Pathog..

[92] Peel AJ, Sargan DR, Baker KS, Hayman DT, Barr JA, Crameri G (2013). Continent-wide panmixia of an African fruit bat facilitates transmission of potentially zoonotic viruses. Nat Commun..

